# Intramedullary cavernous malformation: A case report

**DOI:** 10.1016/j.radcr.2024.08.001

**Published:** 2024-08-31

**Authors:** Nicola Maria Lucarelli, Francesca Troise, Valentina Antonicelli, Sara Greco, Chiara Morelli, Nicola Maggialetti

**Affiliations:** Interdisciplinary Department of Medicine, Section of Radiology and Radiation Oncology, University of Bari “Aldo Moro”, Bari 70124, Italy

**Keywords:** Intramedullary, Spinal cord, Cavernous, Cavernoma, Vascular malformation, MRI

## Abstract

Cavernous malformations are rare vascular anomalies of the central nervous system, occurring in the spinal cord in just 5% of cases. Despite being documented in the literature, intramedullary cavernous malformations are exceedingly rare and often challenging to distinguish from other intramedullary lesions. We report a case of a 42-year-old patient with back pain, right-sided dysesthesias, and impaired proprioception in the distal limbs for approximately 3 months. Magnetic resonance imaging, crucial for differential diagnosis, identified intramedullary cavernous malformations at T11-12. Several conditions can hide the real cause of back pain; however, magnetic resonance imaging can reveal common conditions (such as discal hernia) and rare findings like cavernous malformations. Magnetic resonance imaging remains the study of choice for diagnosing and characterizing intramedullary cavernous malformations.

## Introduction

Cavernous malformations (CMs) are rare vascular anomalies of the central nervous system (CNS), with only 5% located in the spinal cord [1]. Intramedullary cavernous malformations (ICMs) are usually asymptomatic but can present with symptoms such as slowly progressing myelopathy with sensory deficits, lumbar numbness, paralysis below the caudal level, marked weakness, and spasticity in both legs, along with impaired sensation in the lower extremities [2].

Genetic links are present in only 10%-20% of cases, while sporadic CMs may follow previous radiation therapy [3]. ICMs are rarely seen on CT scans, appearing mainly if calcifications are present [2]. MRI is superior in identifying and characterizing these lesions and detecting bleeding. Pathologically, ICMs comprise abnormally enlarged, thin-walled vascular structures without normal medullary tissue between them, predisposing to intramedullary hemorrhage and severe neurological deficits [4].

The typical MRI appearance of a cavernoma includes a “popcorn” or “mulberry” appearance: a well-defined lobulated lesion with a mixed signal core on T1 and T2-weighted images, surrounded by a hypointense rim. This low signal intensity rim is due to hemosiderin and ferritin, while the mixed signal core results from calcification, blood products, fibrosis, and thrombosis. Enhancement is usually absent or very subtle. In smaller lesions, the center appears as a dark dot on T2 and/or on susceptibility-weighted imaging (SWI).

## Case report

**Fig. 1:**
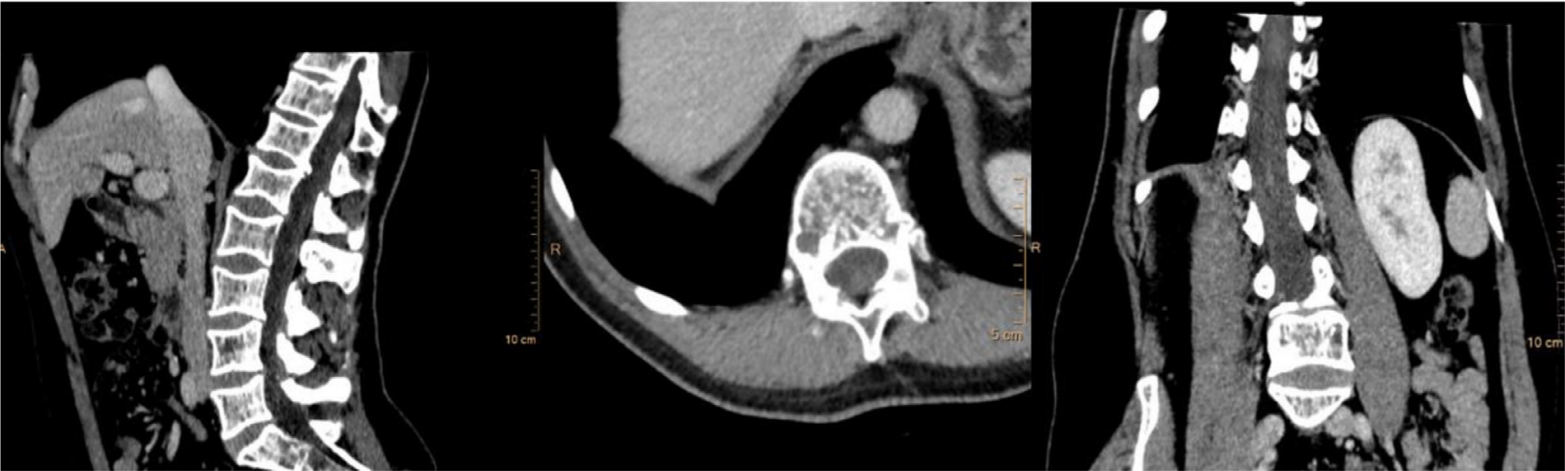
Sagittal, axial, and coronal CT images at the T11-12 level showing the ICM was not visible and no calcifications were present.

A 42-year-old man presented with 2-3 months of right-sided dysesthesias, back pain, and impaired proprioception. Approximately 6 months before, the patient had performed a CT examination due to suspected kidney stones. Upon re-evaluation, no lumbosacral alteration was visible (Fig. 1).

An MRI of the cervical-thoracic-lumbosacral spine was performed due to clinical suspicion of intervertebral disc pathology. The exam utilized TSE sequences with a 1.5 Tesla superconductive device, initially without contrast medium.

**Fig. 2:**
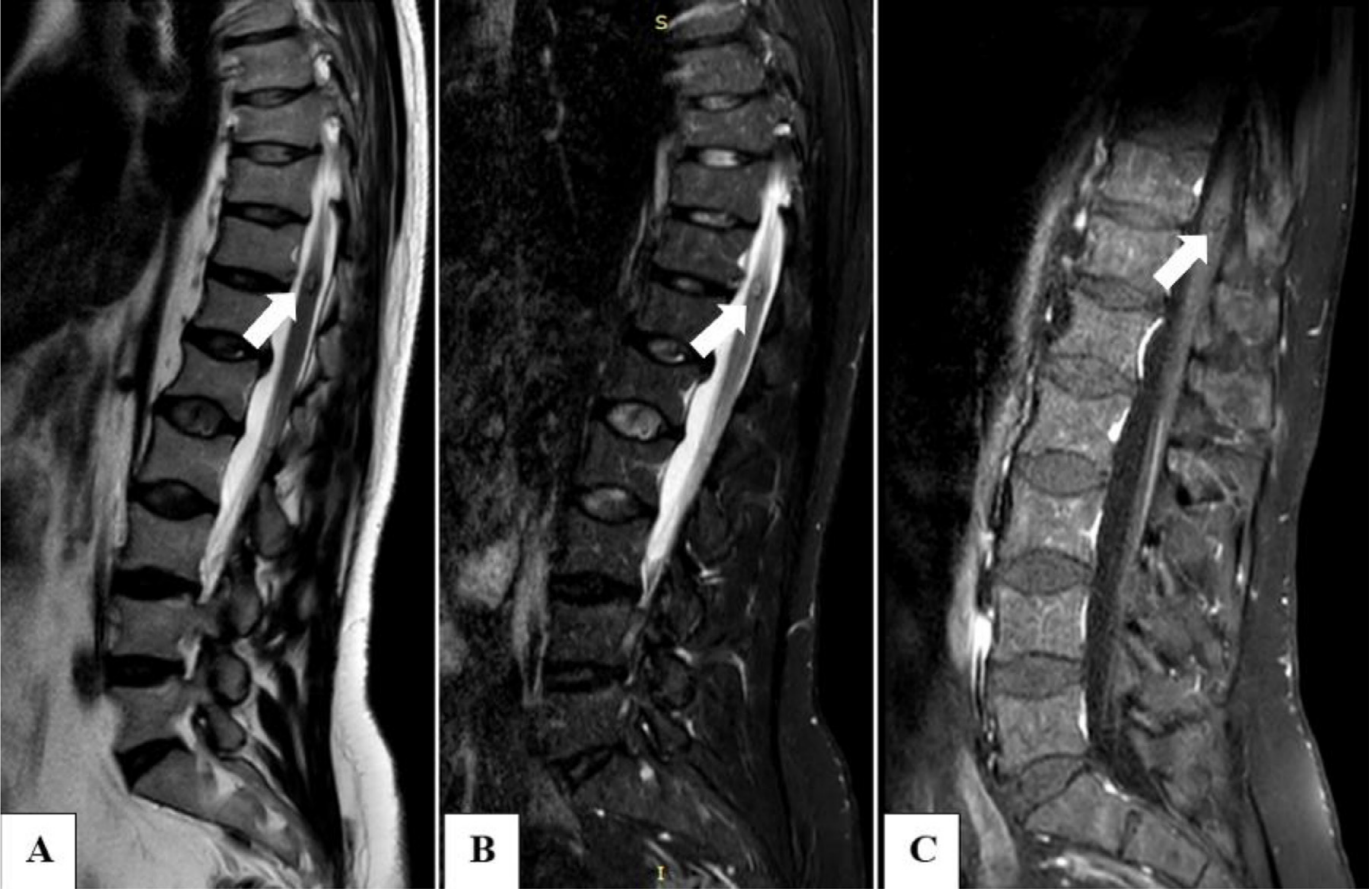
(A) Sagittal T2-weighted MR image showing a well-defined lobulated lesion with a center of mixed signal and a hypointense rim (arrow). (B) Sagittal T2-weighted MR image with fat suppression magnifying the typical ICM characteristics (arrow). (C) Sagittal T1-weighted-SPIR MR image with intravenous contrast material showing slight gadolinium enhancement of the lesion (arrow).

T2-weighted sequences did not show significant discal disease but revealed an intramedullary nodule at T11-12 with a “popcorn” appearance, measuring 11 × 5 mm (CC × LL), without mass effect or perilesional edema. The nodule exhibited a mixed signal with a characteristic hypointense rim of hemosiderin, indicating chronic bleeding, and appeared iso-hypointense on T1-weighted images. These morphological and signal characteristics suggested a cavernoma (Fig. 2).

**Fig. 3:**
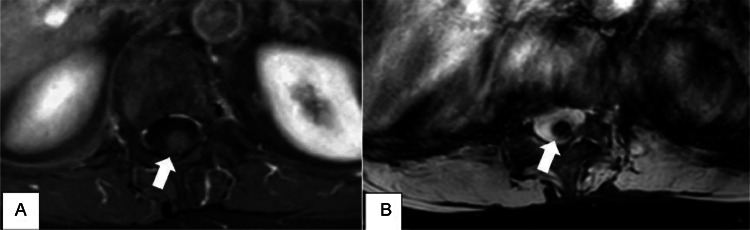
(A) Axial T1-weighted-SPIR MR image with intravenous contrast material showing slight gadolinium enhancement of the lesion (arrow). (B) Axial T2-weighted-FFE MRI image showing a perilesional signal drop due to magnetic susceptibility artefacts, more pronounced in GRE sequences (arrow).

To confirm our suspicions and further study the lesion, a contrast-enhanced MRI was performed using Gadoteric acid (15 mL) and T2*/SWI sequences, crucial for detecting cavernomas due to the magnetic susceptibility of hemosiderin. The results showed an enhanced hypointense rim, lesion inhomogeneity and slight enhancement on T1-weighted images (Fig. 3).

A neurosurgical evaluation recommended total excision of the lesion through a posterior approach with laminectomy and dural opening centered on the pathological zone to completely remove the cavernoma. The patient, however, declined surgical intervention.

## Discussion

We reviewed the literature on intramedullary spinal cord malformations.

The lack of specificity in clinical and imaging features, the difficulty in tissue characterization, and the multitude of differential diagnoses complicate the identification of the lesion on MRI.

As lesions in this critical neurological area can present with similar nonspecific symptoms, MRI is essential for accurate differential diagnosis and prognostication of ICM, as it identifies traces of bleeding.

To rule out an arteriovenous fistula, a spinal cord vascular malformation characterized by a connection between a dural artery and a perimedullary vein, we performed dynamic contrast-enhanced MRI. Early diagnosis with MRI showing intramedullary edema on T2W and markedly dilated superficial veins is crucial to prevent progression and worsening of symptoms [5].

For intramedullary neoplasms, 3 MRI characteristics are noted: focal or diffuse spinal cord expansion, high signal intensity on proton density and T2W, and varying degrees of contrast enhancement. The absence of enhancement does not exclude neoplasms in the presence of cord expansion, a feature not seen in ICM.

Transverse myelitis is another condition with a similar clinical presentation. Its MRI appearance is variable, with typical signal characteristics: T1 isointense or hypointense, poorly delineated hyperintense signal on T2, and variable enhancement patterns (none, diffuse, patchy, peripheral). Differentiation from cavernous angioma is achieved through radiology, clinical information, blood tests, and CSF analysis [6].

Treatment of symptomatic ICM typically involves total excision. The decision for surgery should consider the patient's age, clinical status, and the lesion's size and location [7]. Although no consensus exists, surgical resection is preferred for superficial lesions, with radiosurgery as an option in some cases [8]. Meticulous presurgical planning is essential for optimal outcomes.

## Conclusion

There are various causes of back pain, and MRI is crucial because can identify common conditions (such as discal hernia) and rare findings like cavernous malformations.

The ICM may bleed and the likelihood of recurrent intramedullary hemorrhage increases significantly after the initial presentation with hemorrhage. Neurological function significantly deteriorates after a second bleeding. Thus, early diagnosis is essential for optimal patient management [7,8].

MRI is the method of choice for diagnosing, characterizing, and evaluating the enlargement or bleeding of this lesion.

## Patient consent

Written and signed patient consent was obtained from the patient for publication of this case report.

## CRediT authorship contribution statement

**Nicola Maria Lucarelli:** Writing – original draft, Conceptualization. **Francesca Troise:** Writing – original draft, Conceptualization. **Valentina Antonicelli:** Writing – original draft, Conceptualization. **Sara Greco:** Writing – review & editing, Supervision. **Chiara Morelli:** Writing – review & editing, Supervision. **Nicola Maggialetti:** .
